# Stakeholder Perspectives on Trustworthy AI for Parkinson Disease Management Using a Cocreation Approach: Qualitative Exploratory Study

**DOI:** 10.2196/73710

**Published:** 2025-08-06

**Authors:** Beatriz Alves, Ghada Alhussein, Sara Riggare, Therese Scott Duncan, Ali Saad, David M Lyreskog, Christos Chatzichristos, Ioannis Gerasimou, Stelios Hadjidimitriou, Leontios J Hadjileontiadis, Sofia B Dias, AI-PROGNOSIS Consortium

**Affiliations:** 1 Faculdade de Motricidade Humana University of Lisbon Lisbon Portugal; 2 Department of Biomedical Engineering and Biotechnology Khalifa University of Science and Technology Abu Dhabi United Arab Emirates; 3 Department of Women’s and Children’s Health Uppsala University Uppsala Sweden; 4 AINIGMA Technologies Leuven Belgium; 5 Neuroscience, Ethics & Society (NEUROSEC), Department of Psychiatry University of Oxford Oxford United Kingdom; 6 Department of Electrical Engineering (ESAT), STADIUS Center for Dynamical Systems, Signal Processing and Data Analytics KU Leuven Leuven Belgium; 7 Department of Electrical Computer Engineering Aristotle University of Thessaloniki Thessaloniki Greece; 8 Center of Interdisciplinary Study of Human Perfomance (CIPER), Faculdade de Motricidade Humana University of Lisbon Lisbon Portugal; 9 See Acknowledgments

**Keywords:** Parkinson disease management, artificial intelligence, trust in AI systems, cocreation, advanced care strategies, stakeholder insights, digital health care solutions, artificial intelligence, disease risk, assessment, prognosis

## Abstract

**Background:**

Parkinson disease (PD) is the fastest-growing neurodegenerative disorder in the world, with prevalence expected to exceed 12 million by 2040, which poses significant health care and societal challenges. Artificial intelligence (AI) systems and wearable sensors hold potential for PD diagnosis, personalized symptom monitoring, and progression prediction. Nonetheless, ethical AI adoption requires several core principles, including user trust, transparency, fairness, and human oversight.

**Objective:**

This study aims to explore and synthesize the perspectives of diverse stakeholders, such as individuals living with PD, health care professionals, AI experts, and bioethicists. The aim was to guide the development of AI-driven digital health solutions, emphasizing transparency, data security, fairness, and bias mitigation while ensuring robust human oversight. These efforts are part of the broader Artificial Intelligence-Based Parkinson’s Disease Risk Assessment and Prognosis (AI-PROGNOSIS) European project, dedicated to advancing ethical and effective AI applications in PD diagnosis and management.

**Methods:**

An exploratory qualitative approach, based on 2 datasets constructed from cocreation workshops, engaged key stakeholders with diverse expertise to gather insights, ensuring a broad range of perspectives and enriching the thematic analysis. A total of 24 participants participated in the cocreation workshops, including 11 (46%) people with PD, 6 (25%) health care professionals, 3 (13%) AI technical experts, 1 (4%) bioethics expert, and 3 (13%) facilitators. Using a semistructured guide, key aspects of the discussion centered on trust, fairness, explainability, autonomy, and the psychological impact of AI in PD care.

**Results:**

Thematic analysis of the cocreation workshop transcripts identified 5 key main themes, each explored through various corresponding subthemes. AI trust and security (theme 1) was highlighted, focusing on data safety and the accuracy and reliability of the AI systems. AI transparency and education (theme 2) emphasized the need for educational initiatives and the importance of transparency and explainability of AI technologies. AI bias (theme 3) was identified as a critical theme, addressing issues of bias and fairness and ensuring equitable access to AI-driven health care solutions. Human oversight (theme 4) stressed the significance of AI-human collaboration and the essential role of human review in AI processes. Finally, AI’s psychological impact (theme 5) examined the emotional impact of AI on patients and how AI is perceived in the context of PD care.

**Conclusions:**

Our findings underline the importance of implementing robust security measures, developing transparent and explainable AI models, reinforcing bias mitigation and reduction strategies and equitable access to treatment, integrating human oversight, and considering the psychological impact of AI-assisted health care. These insights provide actionable guidance for developing trustworthy and effective AI-driven digital PD diagnosis and management solutions.

## Introduction

### Background

Neurological disorders are currently the leading cause of disability, with Parkinson disease (PD) emerging as the most common movement disorder with the fastest growing prevalence and disease burden [1]. Moreover, PD resulted in 5.8 million disability-adjusted life years in 2019, an 81% increase since 2000 [2]. Driven by the older adult population and increasing longevity, the number of people with PD is projected to exceed 12 million worldwide by 2040, which has been identified as the PD pandemic [3]. Therefore, the societal and economic burden of PD will escalate unless more effective treatments or means of prevention are identified [4].

Recent advances in digital health technologies offer significant opportunities to improve the quality of care for people with neurodegenerative diseases [5]. In particular, artificial intelligence (AI)–based systems that use wearable sensor data have shown potential in several aspects of PD care, from early diagnosis [6] and classification [5] to progression monitoring [7] and outcome prediction [8,9], ultimately aiming to optimize treatment and prognosis.

However, as AI-based technologies rapidly advance, ethical considerations and human rights must guide the design, development, and deployment to ensure beneficial impacts on public health and medicine [10]. In this regard, the World Health Organization (WHO) outlines 6 key pillars for ethical AI in health care, namely human autonomy, well-being and safety, transparency and explainability, accountability, inclusiveness and equity, and promoting responsive and sustainable AI [11]. These principles align with other frameworks emphasizing legal data processing, human oversight, bias avoidance, safeguarding vulnerable populations, and ensuring patient involvement [12]. For instance, the High-Level Expert Group on AI has established the Assessment List for Trustworthy Artificial Intelligence to self-assess the trustworthiness of AI systems. This framework is based on 7 key requirements, namely human agency and oversight, technical robustness and security, privacy and data governance, transparency, diversity and fairness, societal and environmental well-being, and accountability [13]. Furthermore, the need for patient-centered approaches in digital health is reinforced by Riggare et al [14], who highlighted the perspectives of people with PD regarding symptom management, collaboration with health care professionals (HCPs), and access to relevant health information. This work uniquely emphasized the lived experiences of individuals navigating digital health tools, providing insights into their expectations, needs, and challenges. By focusing on direct patient feedback, the study highlighted the need to integrate user-driven considerations into the development of AI-based health care solutions.

The design process of digital health technologies often overlooks the interplay between technology, users, and their socioeconomic environment [15]. This oversight can impact effectiveness, as evidenced by high attrition rates in digital health research, estimated at 43% in studies involving people living with chronic conditions [16]. In fact, the adoption rate of digital health technologies has decreased significantly over time, with 32% of the users discontinuing their use after 6 months and 50% after more than a year [17].

To address these challenges, research has demonstrated the value of participatory approaches in the design of digital health solutions [16,18]. Cocreation processes actively engage key stakeholders throughout the development process and hold significant potential for improving digital health technologies [19]. A participatory approach is defined by collaboration among diverse participants who contribute with their experiences and expertise across all stages of research, from problem definition to implementation and evaluation [20,21]. Notably, cocreated solutions demonstrate 2.1 times higher long-term adoption rates [10], addressing the sustainability challenges observed in digital health technologies. Moreover, a recent study examined the perspectives of citizens and experts on AI ethics in population health, emphasizing the need for greater public involvement. The findings revealed broad agreement for transparent and accessible ways to include the public in AI decision-making [22]. This aligns with efforts to develop socially responsible data contracts that go beyond legal requirements, such as the “commitments for the digital age,” a cocreated framework that established clear commitments for data stewardship, governance, and accountability in the collection, use, and sharing of personal data [23].

Workshops are considered a key component of the cocreation process, enabling active participation and engagement, allowing individuals to contribute their unique insights, and fostering a sense of ownership. In addition, workshops provide a platform for immediate feedback and iteration, ensuring that solutions are responsive to participants’ input and preferences [24]. This iterative approach has been successfully used in developing mobile health tools, leading to more user-centric and effective interventions [25,26].

Previous studies have also explored participatory approaches to developing digital health solutions for PD. For instance, the eCARE-PD (a virtual coach to support self-care at home) study focused on co-designing a personalized digital companion, engaging people with PD, caregivers, and HCPs to identify key design principles that support self-care and symptom management [18]. The iCARE-PD project expanded on these findings to enhance the usability and acceptance of sensor-based technologies within a community-centered integrated care model, aiming to improve clinical support and long-term engagement [27]. By incorporating structured stakeholder input, both studies [18,27] aimed to bridge the gap between technological innovation and real-world patient needs, reinforcing the necessity of user-driven digital health solutions, an approach strongly advocated by Riggare et al [14]. Moreover, Revenäs et al [28] explored the needs and expectations of people with PD and HCPs that informed a functional eHealth prototype to support PD cocare. Similarly, Kessler et al [29] analyzed feedback from people with PD, carers, and HCPs regarding medical and self-management approaches to improve the quality of life of people with PD. The findings highlighted the need for a collaborative care model that includes patient engagement and comprehensive support.

This study, part of the Artificial Intelligence-Based Parkinson’s Disease Risk Assessment and Prognosis (AI-PROGNOSIS) Horizon Europe research initiative [30], aims to explore the perspectives of key stakeholders, including people with PD, HCPs, AI technical experts, and bioethical experts. The goal is to provide actionable insights for designing ethically sound and trustworthy AI solutions for PD management. These solutions are intended to align with end users’ needs, values, and expectations, which are essential factors for successful implementation and acceptance. Furthermore, the AI-PROGNOSIS project seeks to develop AI-based predictive models for personalized PD risk assessment and prognosis. These models will be informed by digital biomarkers collected from everyday devices, such as smartphones and smartwatches. This innovative approach may enable personalized care management and accurate remote monitoring within a comprehensive digital health ecosystem in the future [30]. This study contributes to this initiative by ensuring that the AI solutions developed are grounded in key stakeholders’ real-world needs and preferences, thereby enhancing their practical relevance and potential impact.

### Objectives

This study aims to explore the perspectives of key stakeholders, including people with PD, HCPs, AI technical experts, and bioethical experts, to provide actionable insights for designing ethically sound and trustworthy AI solutions for PD management. By incorporating stakeholder input, this research intends to identify key ethical considerations, challenges, and opportunities in AI adoption for PD care, ultimately informing the development of user-centered and trustworthy digital health technologies.

## Methods

### Overall Context

**Figure 1:**
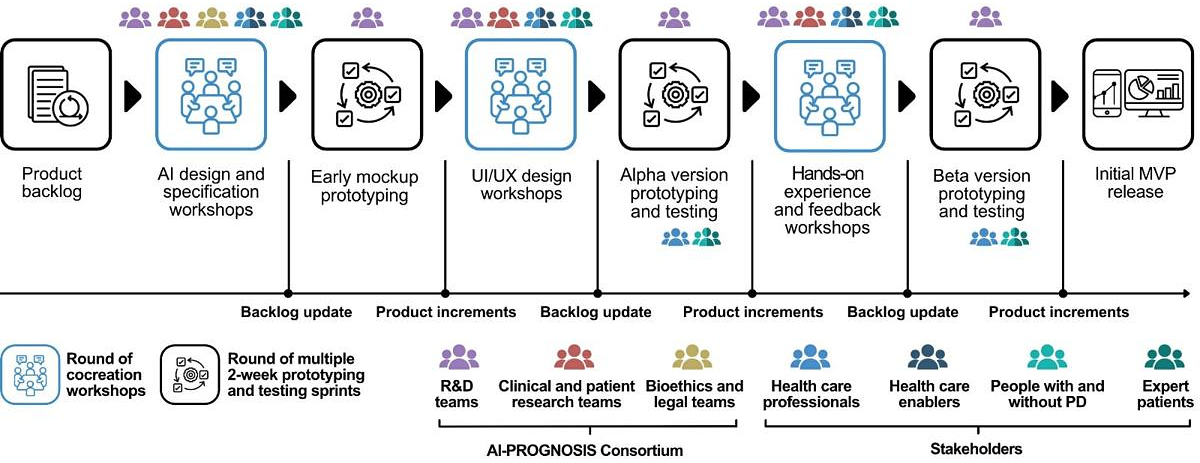
Cocreation approach for the minimum viable products (MVPs) in the Artificial Intelligence-Based Parkinson’s Disease Risk Assessment and Prognosis (AI-PROGNOSIS) project. AI: artificial intelligence; PD: Parkinson disease; R&D: research and development; UI: user interface; UX: user experience.

The AI-PROGNOSIS project aims to design tools that are meaningful, relevant, and meet the needs of users, industry, and society through an iterative collaboration among stakeholders [30]. By using an agile methodology [19,31], the project incorporates 3 rounds of strategic cocreation workshops, as illustrated in Figure 1. These workshops are integral to the development process, ensuring that stakeholder feedback is continuously gathered and integrated into the project’s progression. This qualitative study described the findings of the first round of workshops, which focused on trustworthy AI design and specification. During these workshops, participants engaged in discussions and activities that highlighted the importance of building AI tools that are transparent, secure, and aligned with ethical standards. The insights gained from this initial round of cocreation workshops serve as a foundational step, guiding subsequent iterations and refinements to ensure that the final AI tools are technically robust and socially and ethically sound.

As shown in Figure 1, following the first round of cocreation workshops, a series of prototyping development sprints will update the initial product backlog, focusing on each tool. This process is based on feedback collected during user research and the insights from the first cocreation workshops. These efforts will lead to the first product increment, featuring early mock-up prototyping and internal testing. The second round of cocreation workshops will focus on each tool’s user experience and user interface design to explore the importance and granularity of the insights, metrics, and symptom-tracking features, engaging the various target audiences. Another round of prototyping sprints, moderated and unmoderated usability testing with a small pool of participants, will result in the alpha version of the AI-PROGNOSIS digital tools. The final round of cocreation workshops focuses on hands-on experience and feedback with the tools from all target audiences to identify and refine the features with the highest value and impact potential and set the final technical and product requirements and necessary modifications. The final round of prototyping and testing sprints of the beta versions of the AI-PROGNOSIS tools will produce the initial minimum viable products. Therefore, we plan to expand the pool of recruited participants as we progress toward more extensive alpha and beta testing of the prototypes (including HCPs, health care enablers [eg, policy makers, hospital managers, and heads of PD associations], people with and without PD, and expert patients). Overall, cocreation workshops within the AI-PROGNOSIS project are designed to be conducted both in person and remotely to enhance accessibility. These workshops will use appropriate collaborative tools for various activities, such as brainstorming (Miro), creating interactive mock-ups (Figma), and conducting usability testing (UsabilityHub).

### Study Design

This study adopted an exploratory qualitative design [32] to investigate key stakeholders’ perspectives on integrating trustworthy AI into PD management. In this regard, 4 cocreation workshops were held, each involving different stakeholders. The first 2 workshops included people with PD, and the third and fourth involved HCPs, with all workshops enriched by technical and bioethics experts from the AI-PROGNOSIS consortium. Moreover, each workshop was facilitated by 3 researchers experienced in qualitative methods. They used a semistructured format and open-ended questions to encourage group discussions and elicit participants’ opinions and experiences.

The following key aspects were discussed during the cocreation workshops: (1) factors influencing trust in AI, (2) human autonomy and AI decision-making, (3) fairness and equality, (4) transparency and explainability, and (5) ethical and user-driven concerns around the use of AI in health care and self-care.

### Recruitment

To ensure a diverse representation of perspectives within the AI-PROGNOSIS consortium, a patient panel was established using purposive and snowball sampling [32]. This approach aimed to recruit participants from 6 targeted countries (ie, France, Germany, Portugal, Spain, Sweden, and the United Kingdom) in the AI-PROGNOSIS consortium. We acknowledge that this sampling approach may limit the generalizability of findings, as participants were not randomly selected, and the sampling process may introduce selection bias by disproportionately including individuals with a strong interest in AI and digital health. However, this sampling method is particularly valuable in qualitative research where the focus is on gathering in-depth insights from key stakeholders with specialized knowledge or experience. To mitigate potential biases, efforts were made to include participants from diverse demographics, professional roles, and geographic locations. Once a participant agreed to join the patient panel, informed consent was obtained and recorded verbally. An onboarding session was conducted with each participant via Zoom platform (Zoom Communications, Inc), covering their diagnosis journey, opinions on AI in health care, and expectations regarding the project.

Patient panel members (n=13) were recruited via email from a project member living with PD, who had established a contact network with other people with PD. Inclusion criteria for people with PD were as follows: (1) PD diagnosis, (2) resided in 1 of the 6 targeted countries (ie, France, Germany, Portugal, Spain, Sweden, the United Kingdom), (3) sufficient English proficiency to participate, and (4) had a basic understanding of AI and eHealth. This process aimed to ensure diversity in age, sex, diagnosis and treatment experiences, employment status, and AI knowledge.

HCPs (n=14) were invited via email through professional networks and health care institutions. Inclusion criteria for HCPs were as follows: (1) represented primary (ie, general practitioner) and secondary (ie, neurologist and movement disorder specialist) HCPs in PD care across France, Germany, Spain and the United Kingdom, (2) active involvement in PD care, (3) at least 2 years of clinical experience, (4) sufficient English proficiency to participate, and (5) had a basic understanding of AI in health care.

Technical and bioethical experts, being members of the AI-PROGNOSIS consortium, were used as a convenience sample, with direct informal recruitment. Their expertise significantly enriched the cocreation process, enhancing the quality and depth of the discussions during the workshops.

Individuals who showed interest were provided with detailed project information and invited to an online informative session. Those unable to attend received a session recording and could ask follow-up questions. All participants provided verbal informed consent before the informative sessions, and participation was voluntary.

### Data Collection

Two datasets were created based on data collected through cocreation workshops targeting the perspectives of people with PD (workshops 1 and 2) and the perspectives of HCPs (workshops 3 and 4). These workshops were held in March 2024 and November 2024, respectively. All workshops were conducted online via the Zoom platform and allowed for an in-depth exploration of topics, with the HCP workshop lasting approximately 60 minutes and the workshop targeting people with PD lasting 120 minutes. To ensure comprehensive data capture, both workshops were audio recorded, transcribed verbatim, and pseudoanonymized to maintain confidentiality. Participants were informed of the voluntary nature of the study and assured of the confidentiality of the data collected.

Semistructured scripts guided the workshops with people with PD and HCPs (Multimedia Appendix 1). These scripts included open-ended questions designed to explore participants’ experiences, perceptions, and expectations regarding AI in health care. The topics covered during the workshops are included in Textbox 1.

**Topics covered during the workshops.**

- Diagnosis journeys
- Attitudes toward artificial intelligence (AI) in health care
- Factors influencing trust in AI
- Human autonomy and AI decision-making
- Fairness and equality
- Transparency and explainability
- Ethical and user-driven concerns around the use of AI in health care and self-care

### Data Analysis

**Figure 2:**
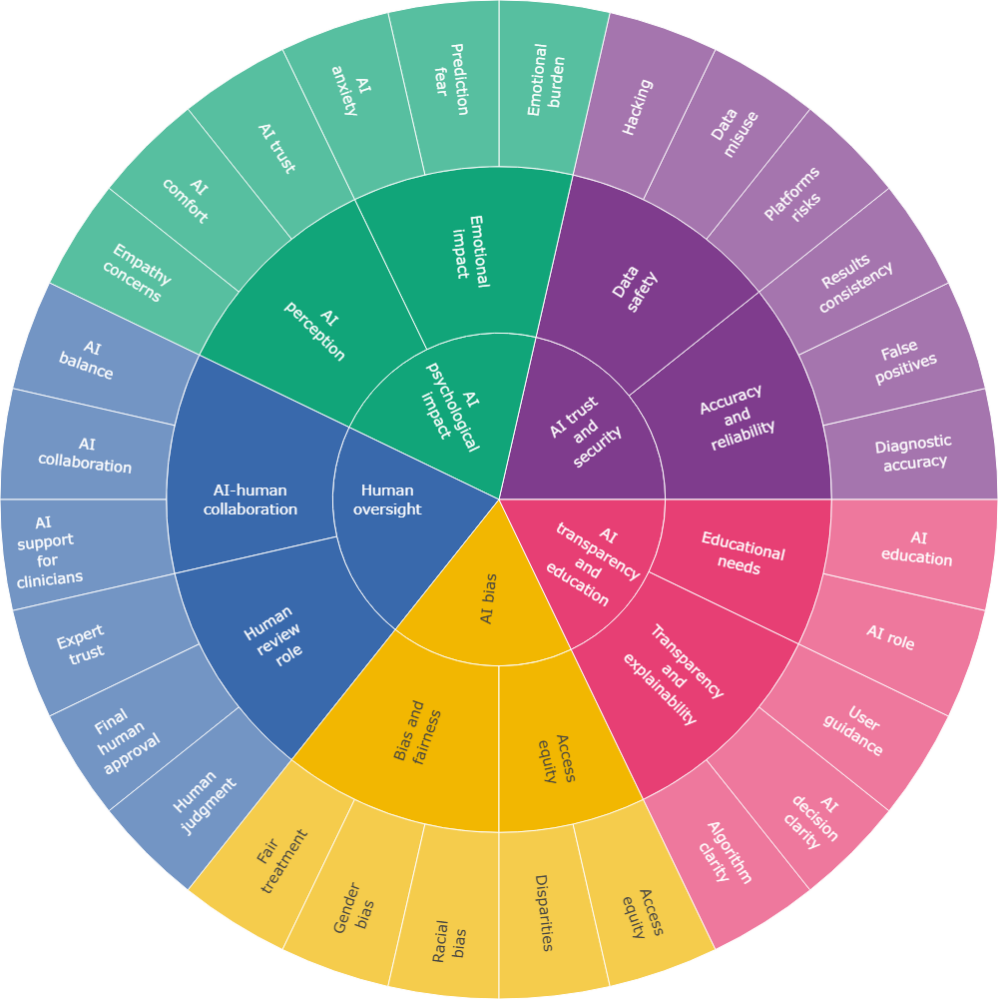
Overview of the main findings derived from the thematic analysis, including themes (inner part of the circle), subthemes (middle part of the circle), and corresponding codes (outer part of the circle). AI: artificial intelligence.

Data analysis followed an inductive thematic analysis process from a holistic perspective of the AI-PROGNOSIS AI-driven ecosystem, with 2 independent researchers (BA and SBD) analyzing all qualitative data. After familiarizing with the data, an initial set of codes was developed (outer circle in Figure 2). These codes were then organized into themes and subthemes, which were reviewed for alignment with the data.

The final themes and subthemes were articulated using clear and precise language, following the methodology outlined by Braun and Clarke [33]. To ensure the credibility and reliability of the analysis, themes were cross-validated. Agreement on code assignment was measured using Cohen κ statistic [34], with a threshold of 0.80 considered acceptable [34]. The final themes were established through consensus building among the researchers. This thorough approach allowed for a comprehensive understanding of the stakeholders’ perspectives, ensuring that the analysis accurately reflected their experiences and insights.

### Ethical Considerations

Ethics approval was granted by the Swedish Ethical Review Authority, Sweden (reference number 2023-05949-01). Participants gave informed consent before participating in the study, and no compensation was provided. They were also informed that their responses would remain anonymous.

## Results

### Research Participants

In terms of workshop participation, 11 people with PD and 6 HCPs attended the first and second workshops, respectively. The HCPs included 2 (33%) general physicians, 2 (33%) neurologists, and 2 (33%) movement disorder specialists. In addition, 3 technical experts and 1 bioethics expert attended both workshops, along with the 3 facilitators and observers. On average, people with PD were aged 54.00 (SD 11.19) years, with 36% (4/11) being female participants, and the average time since diagnosis was 8.4 (SD 5.54) years. On average, the HCPs were aged 43.33 (SD 14.61) years, with 83% (5/6) being male participants, and had an average of 15.00 (SD 14.63) years of experience working with populations with PD (Table 1).

**Table 1:** Demographic characteristics of the study participants.

Participant characteristics	Age (y), mean (SD)	Male participants, n (%)	PD^a^ duration (y), mean (SD)	Experience (y), mean (SD)	Workshop attendance
People with PD (n=11)	54.0 (11.2)	7 (64)	8.4 (5.5)	—^b^	Workshops 1 and 2
HCP^c^ (n=6)	43.3 (14.6)	5 (83)	—	15.0 (14.6)	Workshops 3 and 4
Technical expert (n=3)	35.7 (5.1)	3 (100)	—	6.0 (4.6)	Workshops 1 to 4
Bioethics expert (n=1)	35.0	1 (100)	—	6.0	Workshops 1 to 4

^a^PD: Parkinson disease.

^b^Not applicable.

^c^HCP: health care professional.

### Overview

Regarding the agreement during the process of thematic analysis across all resolution scales (themes and subthemes), the 2 independent researchers (BA and SBD) demonstrated a Cohen κ of 0.95. In cases of uncertainty, discussions between the researchers led to revisions of the text, ultimately reaching 100% agreement on all themes. Moreover, to further reduce potential biases, researchers actively engaged in reflexivity, acknowledging their perspectives and ensuring their interpretations aligned with the participant narratives. A peer debriefing process, as outlined by Janesick [35], was implemented, allowing researchers to critically assess interpretations and refine thematic structures. In addition, consensus building was used to validate findings, ensuring themes were firmly grounded in the data rather than shaped by individual assumptions.

The thematic analysis revealed 5 major themes as follows: AI trust and security (theme 1), AI transparency and education (theme 2), AI bias (theme 3), human oversight (theme 4), and AI’s psychological impact (theme 5). These themes were further detailed through various subthemes (Textbox 2).

The main findings, including themes, subthemes, and corresponding codes, are summarized in Figure 2. These results can help understand the key factors influencing the integration of trustworthy AI into PD management, reflecting the diverse perspectives of stakeholders.

The main findings for each theme and subtheme are briefly presented and supported by direct quotations. Participant identifiers and the respective workshops (workshops 1-4) in which the quotes were provided are included for context.

**Major themes and subthemes from the thematic analysis.**

- AI trust and security (theme 1): This theme included subthemes, such as data safety, accuracy, and reliability. Participants emphasized the importance of ensuring that AI systems are secure and trustworthy to gain user confidence.
- AI transparency and education (theme 2): This theme encompassed subthemes related to educational needs, transparency, and explainability. Stakeholders highlighted the need for clear and accessible information about how AI systems work and their decision-making processes to foster trust and understanding.
- AI bias (theme 3): This theme was examined through subthemes such as bias and fairness and access equity; this theme addressed concerns about potential biases in AI algorithms and the importance of ensuring equitable access to AI-driven health care solutions for all individuals.
- Human oversight (theme 4): This theme was explored through subthemes, including AI-human collaboration and the human review role. Participants discussed the necessity of maintaining human oversight in AI applications to ensure accountability and ethical decision-making.
- AI’s psychological impact (theme 5): Analyzed through subthemes, such as emotional impact and AI perception, this theme highlighted the psychological effects of AI on users, including their feelings of trust, anxiety, and overall perception of AI in health care.

### Theme 1: AI Trust and Security

#### Overview

Integrating AI in the context of PD diagnosis and management raised several concerns about data security and system reliability. Participants recognized the need to implement robust security measures and clear protocols for data handling as a key factor for building trust in AI systems.

#### Subtheme 1: Data Safety

Participants expressed several concerns about data security and integrity in AI systems, highlighting the potential risks associated with insecure platforms and data misuse. In this vein, ensuring robust security measures to protect patient data from breaches and unauthorized access was considered a critical area of focus for AI systems:

In terms of what makes it untrustworthy, you would be having insecure platforms for holding the data...if it was in a cloud that anyone could access or hack into, that would be untrustworthy.

Person with PD 1; workshop 1

HCPs also acknowledged the risks associated with data collection, storage, and access. The importance of restricted access to prevent privacy concerns was highlighted as well as the broader implications of data misuse. This scenario could affect insurance coverage, which illustrates the consequences of inadequate data protection in health care:

There is always a risk in the use of this data, how to collect it, how to store it, and who can access it. This needs to be very clear with strictly restricted access to prevent privacy concerns. ...This data is very strong and could be used by companies to deny patients insurance.

HCP 1; workshop 3

#### Subtheme 2: Accuracy and Reliability

The accuracy and reliability of AI systems were considered crucial factors in building trust among users. The importance of avoiding false positives and ensuring diagnostic accuracy was repeatedly mentioned, considering the unnecessary distress it could cause. A patient with PD emphasized the importance of consistency in AI outputs, and another highlighted the potential consequences of inaccurate results:

To me, a trustworthy system would be a system where if you ask twice the same question, it will come with similar answers, not radically different.

Person with PD 2; workshop 1

If the process isn’t very accurate and it said somebody had Parkinson’s when they didn’t, a false positive, that can be pretty untrustworthy.

Person with PD 1; workshop 1

In addition, HCPs expressed the importance of adopting a balanced approach for AI integration into PD management, acknowledging its potential benefits while recognizing its limitations. As highlighted by a participant, clinicians should be properly educated about AI systems to ensure their effective application in clinical practice:

We need to understand how AI works, the data it is based on, and how to interpret its decisions. ...We should not trust it 100%, but neither should we ignore the additional benefits it provides.

HCP 6; workshop 4

### Theme 2: AI Transparency and Education

#### Overview

Transparency and education in AI systems were considered essential for making their processes clear and accessible to all relevant stakeholders. In this regard, participants emphasized that these aspects were vital not only for improving understanding and accessibility but also for addressing misconceptions and building confidence in AI-driven solutions.

#### Subtheme 1: Educational Needs

Many participants recognized the need for educational resources to help people with PD and HCPs understand AI systems and their role in PD diagnosis and management. This was considered relevant in counteracting negative perceptions and fostering informed engagement with AI-driven health care solutions:

There must be a support educational program to inform what AI actually is because the generic opinion about it at the moment is negative.

Person with PD 3; workshop 1

Furthermore, HCPs recognized the importance of continuous education on AI advancements, acknowledging that empowered clinicians are more likely to view AI as a supportive tool rather than a replacement, as follows:

Doctors’ education must improve to keep up with AI, so they can explain AI-derived solutions to patients. This is an exciting time for evolution in medicine, but everyone needs to be on the same page to provide high-quality care.

HCP 4; workshop 4

Doctors normally are very against things we don’t see. ...If we understand how AI works, we can demystify it. AI shouldn’t be seen as something unreachable and incomprehensible.

HCP 2; workshop 3

#### Subtheme 2: Transparency and Explainability

Overall, participants valued transparency in AI decision-making processes, advocating for the development of explainable AI. Understandable and accessible AI decisions could promote trust and acceptance among their users. In this regard, technical experts from the AI-PROGNOSIS consortium added the following insights:

We should develop Explainable AI...so that the user can understand, for example, what data went into training a model, why was the data chosen, and did we assess the fairness?

Technical AI expert 1; workshop 1

In AI-PROGNOSIS, we are developing models related to the risk prediction of Parkinson’s disease and the progression, and medication response model. All of them are AI driven, we are aiming to implement models that are not just accurate, but they are trustworthy in a sense of they should have some kind of privacy fairness, closeness, generalization explainability so that our model become trustworthy.

Technical AI expert 3; workshop 2

Furthermore, HCPs noted that receiving AI outputs in an easily interpretable format could not only aid clinical decision-making but also lessen skepticism toward unfamiliar technology. One HCP suggested the following:

It should be standardized [i.e., figures, graphics] to improve understanding for non-neurologists, so we don’t need to interpret different explanations, but rather have consistent information.

HCP 3; workshop 3

### Theme 3: AI Bias

#### Overview

Participants expressed significant concern about bias in AI systems, emphasizing the need for equitable treatment and robust bias mitigation strategies. They highlighted examples of racial and gender bias in health care algorithms and discussed the challenges posed by incomplete datasets and variability in clinical assessments.

#### Subtheme 1: Bias and Fairness

Concerns about bias in AI models were particularly prevalent among participants, who mentioned examples of racial and gender bias in existing health care algorithms. One technical expert noted the following:

In the United States, for example, there are health care algorithms that have shown racial bias...the model prioritized patients for high-risk care management programs based on race.

Technical AI expert 2; workshop 1

The challenge of data collection and its impact on AI model development was also discussed. More specifically, time constraints in clinical practice posed a challenge for a comprehensive assessment and were often associated with incomplete datasets, as acknowledged by one HCP who stated the following:

Missing data can be a significant issue, if physicians don’t have enough time to systematically ask about all symptoms, especially nonmotor symptoms.

HCP 6; workshop 4

In this line, one HCP suggested the implementation of wearable-based remote assessments to address this limitation, emphasizing the practicality of this approach, as follows:

We don’t do the complete Movement Disorder Society–Unified Parkinson’s Disease Rating Scale [MDS-UPDRS] at each visit. If you want to do it systematically, you need to use something in an ambulatory setting, like an iPhone or a smartwatch, to continuously record data at home. It’s not feasible to do this [MDS-UPDRS] during clinic visits because, honestly, we don’t have the time.

HCP 2; workshop 3

Moreover, the assessment variability of clinical outcomes could further induce bias in AI models, which must be trained on consistent and reliable data to provide accurate predictions, as suggested by one HCP:

The most challenging values to be gathered by AI would be the normal variability in the physical exam. The pull test is so variable...we should find a more reliable way to establish balance. Besides, tremor, rigidity, and bradykinesia are very difficult to measure consistently.

HCP 4; workshop 4

#### Subtheme 2: Access Equity

Some participants suggested that AI could enhance fairness in medical diagnoses by eliminating human prejudices that might influence decision-making. This highlighted the potential of AI to provide more equitable health care by ensuring consistent and impartial decisions:

AI will bring more fairness to the diagnosis because it’s an algorithm, so it works equally for everybody. There is no prejudice.

Person with PD 2; workshop 1

Nonetheless, the bioethics expert highlighted the complex nature of bias in AI systems and the need for continuous monitoring to address potential biases:

A lot of times when we talk about fairness in AI, it’s about mitigating bias in the algorithms of the model that you build for the AI.

Bioethics expert; workshop 2

Furthermore, patient autonomy and informed consent are crucial to ensuring the ethical use of AI in health care. These aspects are particularly relevant in the case of AI gathering data that patients may not disclose to physicians, and analysis of such data could uncover sensitive information, as noted by one HCP:

AI could gather data from many sources and make connections that the patient never disclosed to the doctor. This could be very dangerous.

HCP 4; workshop 4

### Theme 4: Human Oversight

#### Overview

Participants emphasized the role of human oversight in AI-assisted health care and the relevance of human judgment, accountability, and empathy in clinical decision-making. Discussions highlighted the need for AI to complement rather than replace HCPs, ensuring a collaborative approach that preserves trust, provides reassurance, and supports physician-patient relationships.

#### Subtheme 1: AI-Human Collaboration

Overall, patients with PD strongly preferred AI systems to be overseen by HCPs. This involvement was considered essential for maintaining trust and safeguarding an empathetic therapeutic relationship with HCPs. This perspective showed the need for clear, effective communication from HCPs regarding AI decision-making processes. A patient with PD highlighted a common concern about the potential impersonal nature of AI:

It’s always good to know that someone is supervising the decision; you’re not being treated only by a machine....I think for many people, it would be very scary to know that the health decisions are made by a machine, not a human. It will have to be very well explained.

Person with PD 2; workshop 1

Moreover, HCPs emphasized the relevance of human judgment and accountability in AI-assisted decision-making. This reinforced the common perception that while AI can add value to current diagnostic methods, the final interpretation and responsibility lie with the clinician, as stated by one participant:

The doctor has the final responsibility, and AI is just a tool—like an MRI or CT scan. You would never blame the CT machine, but you would always hold the doctor responsible for interpreting it.

HCP 3; workshop 3

#### Subtheme 2: Human Review Role

Several participants acknowledged that human oversight added a layer of reassurance and accountability, which increased their trust in AI-driven solutions. As expressed by one participant, patients are more likely to trust and engage with technology when human judgment overlays AI systems:

Yeah, I tend to think that AI is getting more and more accurate results, I would give more trust to the to the AI decisions overtime. We tend to give more trust in a human health care professional.

Person with PD 4; workshop 2

Although human oversight was considered essential in maintaining trust and ensuring effective patient care, HCPs recognized the value of AI integration in their practice. This collaborative effort can help mitigate the risks associated with overreliance on AI while still benefiting from its output, as expressed by one participant:

We can use AI to double-check things, but if something seems off, we shouldn’t follow it blindly.

HCP 2; workshop 3

Nonetheless, one HCP noted that AI should not be imposed on clinicians without their consent or understanding, reflecting the need for clear implementation guidelines in clinical settings:

I think the only way AI should be accountable is if it is imposed by your employer, for example, if AI is included in the algorithm of a clinical history at the hospital.

HCP 4; workshop 4

### Theme 5: AI’s Psychological Impact

#### Overview

Participants emphasized the psychological consequences of AI integration in PD care, particularly the potential distress caused by AI-generated diagnoses. There were concerns regarding the risks of false positives, the emotional burden of AI predictions without actionable outcomes, and the fear of losing the human aspect of health care.

#### Subtheme 1: Emotional Impact

Participants voiced some concerns about the potential psychological consequences of AI predictions, particularly regarding the anxiety and stress that might result from AI-generated diagnoses. The risk associated with inaccurate predictions, as highlighted by one participant, illustrated the psychological effects of unreliable AI assessments:

If somebody who might have a diagnosis of Parkinson’s is then told there’s a 75% chance you have got Parkinson’s, that could cause some quite disastrous potentially fatal consequences for an individual who fears that their life is over.

Person with PD 1; workshop 1

Moreover, HCPs stressed the need for thoughtful communication of AI results, discussing the relevance of sharing information that may not lead to actionable outcomes:

We want to predict if someone will develop PD, but if we can’t change the outcome, what is the goal of giving this news now? It’s a hard thing to take in.

HCP 2; workshop 3

#### Subtheme 2: AI Perception

Beyond technical accuracy, attitudes of patients with PD toward AI tools were influenced by psychological factors, including fear and uncertainty about these technological advancements. One patient with PD underlined this perspective, evidencing the need to address existing misconceptions about AI to improve acceptance and trust:

I think there’s a lot of fear about AI. And I think it’s worth considering where AI is already used, for example for auto segmentation or an MRI scan.

Person with PD 2; workshop 1

In addition, HCPs highlighted their role in interpreting and communicating AI outputs in clinical consultations, ensuring that the human aspect of care is not lost in the integration of technology:

One of the main roles of a doctor is to guide the patient through their illness, to explain what they are feeling, what they are going through, and to be with them during the process.... You can use AI as a tool to find the best option, but trust comes from your ability to explain why you’re choosing one solution over another.

HCP 4; workshop 4

Another HCP also reinforced the importance of actively involving patients in shared decision-making processes:

We need to compare the AI data with clinical data and make decisions together with the patient. This approach could increase patient trust in our decisions as they can see the data and understand how we interpret and use it in clinical practice.

HCP 1; workshop 3

Overall, these findings suggested that stakeholders’ trust in AI-driven solutions for PD diagnosis and management relied on data security, accuracy, transparency, and human oversight. Concerns about data breaches, biases, and the psychological impact of AI predictions highlighted the need for AI tools to be secure, fair, explainable, and sensitive to mental health. Addressing these factors is essential to gain patient acceptance and trust in clinical practice.

## Discussion

### Principal Findings

#### Overview

Grounded by a cocreation approach, this study explored key stakeholders’ perspectives on the challenges and opportunities of integrating trustworthy AI into PD diagnosis and management. As a participatory approach, the added value of cocreation lies in capturing lived experiences and end-user empirical knowledge to ground innovative solutions’ development [16]. These efforts aim to promote the acceptability, usability, and effectiveness of the resulting digital technologies, ensuring user satisfaction [19,31].

Driven by the aforementioned perspectives, 4 cocreation workshops were conducted, actively engaging key stakeholders (ie, people with PD, HCPs, and technical and bioethics experts). Thematic analysis of the transcripts revealed 5 main themes: AI trust and security (theme 1), transparency and education (theme 2), AI bias (theme 3), human oversight (theme 4), and AI’s psychological impact (theme 5), as discussed subsequently.

#### AI Trust and Security

Overall, participants expressed significant concerns about data privacy and the potential misuse of sensitive health information. Particularly, people with PD underlined the risks associated with insecure platforms and unauthorized access, while HCPs emphasized the need for robust data storage to mitigate privacy risks. This reinforces the argument that trust in AI systems is influenced by users’ perceptions of AI capabilities and input data quality. However, AI models require large datasets for more accurate outputs, which can lead to privacy issues if not managed properly [36]. These insights contribute to the broader field of AI trust, risk, and security management, highlighting the need for robust data security measures and consistent accuracy in AI systems to build patient trust and ensure effective implementation [37]. Moreover, Reinhardt [38] identified several dimensions of trust in AI, namely interpersonal trust, institutional trust, and trust in technology, emphasizing that the current understanding of trust and trustworthiness in AI ethics lacks a clear operational framework. In this vein, the WHO [11] also emphasizes that ethics and human rights must be at the heart of AI design, deployment, and use to maximize benefits and minimize risks. This includes ensuring robust security measures to protect patient data and foster confidence among stakeholders.

#### Transparency and Education

Many participants highlighted the need for clear communication about AI’s functionality and decision-making processes. Ethical guidelines mandate clear explanations of AI systems, ensuring they are technically sound and ethically responsible [39]. In addition, people with PD stressed the importance of demystifying AI through accessible educational resources, which aligns with research suggesting that clear explanations of AI processes promote trust by helping users understand its behavior, reducing fear and skepticism associated with unfamiliar technology [40]. Furthermore, HCPs highlighted the relevance of explainable AI, aligning with the concepts discussed in the literature, aiming to make AI models more interpretable [41,42]. The successful integration of AI in health care requires educated and empowered clinicians who view AI as a supportive tool rather than a replacement for clinical expertise [40,43]. In addition, the WHO report [11] advocates for educating stakeholders on the function of AI systems, including their strengths and limitations, to encourage informed decision-making among health care providers and patients.

#### AI Bias

In general, participants raised concerns about bias in AI models, particularly racial and gender disparities. This illustrates the concept of algorithmic bias, which can result in discriminatory outcomes by making decisions based on biased data [44]. The results of the study by Abràmoff et al [45] reinforced the impact of racial and gender biases in AI health care algorithms, highlighting the need for equitable treatment and robust bias mitigation strategies. Moreover, HCPs pointed out the challenges of incomplete datasets due to time constraints in clinical practice and the risks of biased data influencing AI predictions. Data bias, originating from unrepresentative datasets, can lead to skewed model predictions [44]. Continuous data collection technology, as recommended by one HCP, aligns with current guidelines for enhancing AI research quality by incorporating diverse data sources and methodologies [46]. In addition, variability in clinical outcome assessments poses a challenge for AI models, which require consistent and reliable data for accurate predictions. Feeding biased data into AI systems can result in systematically biased outputs and discriminatory practices [47]. Hence, transparency about data sources and clear distinctions between efficacy and effectiveness are essential to prevent, identify, and correct these issues. This aligns with a systematic review of neuroimaging-based AI in PD, emphasizing the importance of addressing data imbalance and using robust methodologies to improve AI quality and applicability in clinical settings [46].

#### Human Oversight

Overall, patients with PD strongly prefer human oversight, which builds trust and ensures empathetic care. Conversely, HCPs emphasized balancing AI’s benefits with human expertise to alleviate concerns about AI’s impersonal nature and promote accountable, transparent decision-making. This supports the view that AI should support rather than replace clinical decision-making [36,43]. The preference for human oversight in AI-driven health care aligns with the findings of the study by Laux [48], highlighting its role in addressing institutional distrust toward AI and supporting democratic design principles in AI governance. While AI adds value to diagnostic methods, the final interpretation and responsibility lie with clinicians, ensuring patient safety and effective care. Clear implementation guidelines are needed to ensure that AI is not imposed on clinicians without their consent or understanding, as successful adoption requires clinician buy-in and a comprehensive understanding of AI’s benefits [43]. Balancing technology and human expertise, as highlighted by HCPs, helps mitigate risks associated with overreliance on AI while benefiting from its outputs. Although AI can free up time for clinical consultations, it may limit clinicians’ ability to explain decisions, making human oversight crucial [49]. In addition, reliance on AI could lead to gaps in treatment quality for patients who withhold data, creating conflicts between patient consent and the standard of care [50].

#### AI’s Psychological Impact

Many participants highlighted concerns about the emotional toll of AI predictions. Particularly, people with PD expressed anxiety and distress caused by false positives or premature diagnoses. Both people with PD and HCPs expressed concerns that predictions of developing PD should not be communicated to people at risk, because there is nothing to be done to change the outcome. Schaeffer et al [51] describe skepticism among people with PD regarding early detection and disclosure of risk. However, early detection and disclosure may be considered acceptable if the individual wishes to receive this information, provided there are suggested lifestyle changes based on the prediction and regular follow-up of individuals afterward. Furthermore, HCPs reinforced the need for sensitive communication of AI-generated results to minimize psychological harm and ensure patient well-being. Thoughtful communication from HCPs regarding the use of AI technologies could alleviate patient concerns while reaffirming the supportive role of these advancements in PD care, as discussed by Ha and Kim [40] and Godoy Junior et al [43]. In this regard, Asan et al [36] suggested that AI outputs must be presented in a way that is sensitive to the patient’s emotional and psychological needs, balancing technological innovation with ethical and compassionate care. Clinicians’ ability to communicate their rationale behind clinical decisions improves patient understanding and confidence in the treatment approach [43]. This perspective underlines the need for clinicians to be educated to interpret and communicate AI outputs, aiming to improve patient engagement and satisfaction [40]. In this way, patients are more likely to trust the health care system and clinical decisions when they are actively involved in their care [43]. Addressing patient mental health in the development and implementation of AI tools in health care is crucial. Ensuring thoughtful communication of AI results can reduce stress and support a more human-centered approach to AI integration. In this regard, De Freitas et al [52] suggested that patients’ attitudes toward AI tools are significantly influenced by psychological factors, such as fear of inaccurate predictions and the emotional impact of AI-generated results. Addressing these psychological concerns seems crucial for improving patient acceptance and trust in AI technologies.

### Implications

This exploratory qualitative study offers valuable insights regarding stakeholder perspectives on the development and implementation of AI-based digital solutions in PD diagnosis and management. From a theoretical standpoint, these findings contribute to a framework for trustworthy AI in PD care, as evidenced by the interdependencies among the emerging themes. For instance, addressing AI bias enhances trust, while effective education supports meaningful human oversight. By considering these aspects, stakeholders can work toward patient-centered AI solutions that are both technically sound and ethically robust. Furthermore, the emphasis on interdisciplinary collaboration, as evidenced by the diverse group of participants, stresses the value of adopting cocreation methodologies in this context. Considering the complex interplay of motor and nonmotor PD symptoms, gathering input from key stakeholders is particularly relevant in PD care to develop comprehensive AI-driven solutions, aligning with recent trends in participatory design for health technologies [20,25].

Building on these theoretical insights, several practical implications can also emerge from the identified themes. The “AI trust and security” theme reinforces the need for robust security measures and trustworthy algorithms in AI applications for PD care. This is particularly relevant considering the sensitive nature of the data, which may include detailed motor assessments and personal health information. Research suggests that users are more likely to engage with technologies they perceive as secure [53], aligning with findings from studies on trust in AI-based health information technologies [36,38].

Moreover, the “AI transparency and education” theme highlights the importance of developing educational resources to promote trust and acceptance among users. These results are consistent with the findings of Dias et al [54], suggesting that tailored educational resources improve technology acceptance in the context of wearable neurofeedback for students’ stress and anxiety management. Consequently, these findings could inform the development of ethical guidelines and policies governing AI deployment in PD care settings, providing actionable insights for policy makers and health care organizations.

From a managerial perspective, the “AI bias” theme reinforces the need for protocols to ensure fair and equitable access to AI-driven PD care across diverse patient populations. Health care organizations should implement bias mitigation strategies in AI systems, such as diverse data collection and regular algorithmic audits, to address disparities in AI model performance across patient subgroups [55]. This is particularly important in PD care, where age and gender biases in symptom recognition could impact diagnosis and the course of treatment [46].

The “human oversight” theme emphasizes the critical role of HCPs in AI integration. Considering the pathophysiology of PD, with symptom fluctuations and variable treatment responses, human oversight is crucial for interpreting AI outputs in the context of individual patient needs. Therefore, organizations should develop clear protocols for AI-human collaboration, including defined roles for HCPs in reviewing and interpreting AI outputs, to effectively integrate AI into clinical workflows while maintaining accountability.

Finally, the “AI’s psychological impact” theme stresses the need for support systems addressing the emotional aspects of AI interaction in PD care. The support system could involve developing AI interfaces that are sensitive to cognitive and emotional states common in PD, such as anxiety, depression, or cognitive fluctuations. In this context, the MoveONParkinson study [56] pointed out the relevance of incorporating behavior change and self-management principles into digital solutions for PD care. In line with these findings, AI interfaces could be designed to include conversational agents that provide personalized support and encourage patient autonomy. This approach was implemented by Macedo et al [57], outlining the potential of conversational agents in providing personalized support and motivation for exercise adherence in people with PD. Furthermore, the user-centered design principles used in these studies align with adopting a cocreation approach in this study, highlighting the relevance of stakeholder engagement in this field. By ensuring AI systems are not only technically sound but also acceptable, usable, and responsive to the needs of people with PD, we can potentially improve engagement, reduce anxiety, and improve the experience of people with PD with AI-driven care solutions.

Overall, the main findings presented in this study are aligned with WHO ethical guidelines [11] while extending their application through stakeholder-driven perspectives. From a theoretical standpoint, this study reinforces the importance of trust in AI, linking it to security, transparency, and explainability [37]. It highlights how user-centered design and ethical AI principles can address challenges related to algorithmic bias and human oversight [38,41]. From a policy perspective, the findings emphasize the need for comprehensive regulations, including bias mitigation, equitable access, and clinician involvement in AI decision-making [45,48]. In fact, policy makers should prioritize education programs to foster trust and acceptance in line with WHO ethical guidelines [11]. By bridging theoretical advancements with actionable policy recommendations, this study extends its relevance beyond the immediate findings, providing a blueprint for future development and implementation of AI solutions in PD care. These broader implications can validate the findings and situate them within the ongoing discourse on responsible AI governance and human-centered digital health innovation.

Furthermore, the cocreation workshops provided valuable stakeholder insights into developing trustworthy AI solutions in PD management but revealed areas for improvement in future iterations. One key challenge was uneven engagement among stakeholder groups; while people with PD were highly active, technical experts and bioethicists contributed less frequently, potentially limiting interdisciplinary discussions on ethical and technical concerns [24]. In addition, logistical complexities in web-based workshops, such as unstable internet connections and varying digital literacy levels, affected interaction quality. These obstacles align with the findings of Dugstad et al [26], who noted that virtual formats may hinder spontaneous collaboration compared to in-person settings. Another challenge was translating stakeholder feedback into actionable design features, as synthesizing diverse perspectives required significant effort. This reflects broader issues in cocreation processes, where balancing inclusivity and feasibility presents difficulties [19]. Finally, this study highlights the importance of iterative feedback to enhance stakeholder alignment, ensuring AI solutions remain relevant and user centered. Literature on participatory design supports continuous validation to refine AI-based health care interventions [20].

### Limitations

While this study provides valuable insights, there are a few limitations to consider. The small sample size and specific demographic characteristics may not fully represent the broader stakeholder population, yet they offer a focused perspective. The voluntary nature of participation could have introduced a form of engagement bias, as participants might have been more informed and enthusiastic about AI. In addition, the requirement for English proficiency and a basic understanding of AI could have led to some selection bias. Nonetheless, these criteria ensured that participants could fully engage in the discussion, contributing meaningfully to the findings.

### Future Research

This study described the findings of the first round of cocreation workshops within the AI-PROGNOSIS project approach. Following this, several prototyping sprints are planned to update the product backlog based on user feedback and workshop insights, leading to early mock-ups and internal testing. The second round of workshops will focus on user experience and user interface design, exploring insights, metrics, and symptom-tracking features with target audiences, resulting in alpha versions through usability testing. The final round will gather hands-on audience feedback to refine features and set final requirements, resulting in beta versions and initial minimum viable products. This integrated process of cocreation activities would allow the optimization of the tools produced by the AI-PROGNOSIS project and would thus provide smooth integration of the stakeholders’ voices, incorporating ethical considerations in all aspects of AI use. Further validation and insights are anticipated from the planned extension of the sample size to larger groups, for example, via a survey of the actual users of the AI-PROGNOSIS ecosystem across Europe. Future research could explore key cultural and national differences in perspectives on trustworthy AI for PD management. These variations may stem from distinct health care systems, societal values, and historical experiences with digital health technologies, highlighting the need for context-aware AI solutions. Therefore, future studies should conduct a deeper analysis of these differences to ensure AI-driven health care solutions align with the values and regulatory frameworks of diverse populations. This approach could foster greater acceptance, trust, and usability, reinforcing AI-PROGNOSIS’s commitment to developing culturally adaptive and ethically sound AI tools. In addition, future studies should also explore caregiver engagement strategies, assess their experiences with AI-enabled tools, and examine how AI interfaces can be designed to support both people with PD and caregivers in managing AI-assisted health care effectively. In addition, future qualitative studies could deepen understanding of AI’s psychological impact on people with PD, examining emotional distress from AI-generated predictions, the burden of uncertainty, and the effects of false positives. Research could also explore autonomy concerns, assessing whether AI reliance reduces self-confidence in personal health management. Moreover, cognitive burden may arise from complex AI-driven health care decisions, requiring a user-friendly design to prevent decision fatigue. Addressing these factors could also help shape AI solutions that are both clinically effective and psychologically supportive, fostering trust and engagement in PD care. Comorbidities, such as cardiovascular diseases and diabetes, may play a crucial role in shaping AI adoption, influencing both patient and clinician decision-making processes. Future studies could also examine the interplay between AI applications and comorbidity management, providing deeper insights into the development of personalized, patient-centered AI-driven health care solutions for people with PD. By integrating broader health profiles into AI models, researchers can ensure that AI tools are both clinically effective and responsive to the diverse health needs of people with PD.

### Conclusions

This study offers valuable insights into stakeholder perspectives on integrating AI into PD diagnosis and management. Primary concerns include the need for robust data security to protect sensitive information, the risk of perpetuating bias and fairness issues within AI systems, and the necessity for transparency in AI decision-making processes. In addition, people with PD emphasize the importance of human oversight to complement AI’s support and worry about the potential psychological impact on their sense of autonomy and human connection. Addressing these issues is crucial for developing AI systems that are secure, fair, transparent, and supportive of patient well-being. Focusing on these areas can enhance the trust and efficacy of AI in health care, ultimately improving patient outcomes and satisfaction. By addressing these concerns and leveraging AI’s benefits, AI can be ethically and effectively designed and implemented to improve PD care.

## Appendix

Multimedia Appendix 1 Semistructured guide scripts for cocreation workshops conducted with people with Parkinson disease and health care professionals.

## Abbreviations

AI: artificial intelligence
AI-PROGNOSIS: Artificial Intelligence-Based Parkinson’s Disease Risk Assessment and Prognosis
HCP: health care professional
PD: Parkinson disease
WHO: World Health Organization

## Data Availability

The datasets generated or analyzed during this study are not publicly available due to ethical restrictions and institutional policies but can be obtained from the corresponding author upon reasonable request.

## References

[1] GBD 2016 Parkinson's Disease Collaborators (2018). Global, regional, and national burden of Parkinson's disease, 1990-2016: a systematic analysis for the Global Burden of Disease Study 2016. Lancet Neurol.

[2] Schiess N, Cataldi R, Okun MS, Fothergill-Misbah N, Dorsey ER, Bloem BR, Barretto M, Bhidayasiri R, Brown R, Chishimba L, Chowdhary N, Coslov M, Cubo E, Di Rocco A, Dolhun R, Dowrick C, Fung VS, Gershanik OS, Gifford L, Gordon J, Khalil H, Kühn AA, Lew S, Lim S, Marano MM, Micallef J, Mokaya J, Moukheiber E, Nwabuobi L, Okubadejo N, Pal PK, Shah H, Shalash A, Sherer T, Siddiqui B, Thompson T, Ullrich A, Walker R, Dua T (2022). Six action steps to address global disparities in Parkinson disease: a World Health Organization priority. JAMA Neurol.

[3] Dorsey ER, Sherer T, Okun MS, Bloem BR (2018). The emerging evidence of the Parkinson pandemic. J Parkinsons Dis.

[4] Chaudhuri KR, Azulay JP, Odin P, Lindvall S, Domingos J, Alobaidi A, Kandukuri PL, Chaudhari VS, Parra JC, Yamazaki T, Oddsdottir J, Wright J, Martinez-Martin P (2024). Economic burden of Parkinson's disease: a multinational, real-world, cost-of-illness study. Drugs Real World Outcomes.

[5] Templeton JM, Poellabauer C, Schneider S (2022). Classification of Parkinson's disease and its stages using machine learning. Sci Rep.

[6] Sun YM, Wang ZY, Liang YY, Hao CW, Shi CH (2024). Digital biomarkers for precision diagnosis and monitoring in Parkinson's disease. NPJ Digit Med.

[7] Adams JL, Kangarloo T, Gong Y, Khachadourian V, Tracey B, Volfson D, Latzman RD, Cosman J, Edgerton J, Anderson D, Best A, Kostrzebski MA, Auinger P, Wilmot P, Pohlson Y, Jensen-Roberts S, Müller ML, Stephenson D, Dorsey ER (2024). Using a smartwatch and smartphone to assess early Parkinson's disease in the WATCH-PD study over 12 months. NPJ Parkinsons Dis.

[8] Sieberts SK, Borzymowski H, Guan Y, Huang Y, Matzner A, Page A, Bar-Gad I, Beaulieu-Jones B, El-Hanani Y, Goschenhofer J, Javidnia M, Keller MS, Li YC, Saqib M, Smith G, Stanescu A, Venuto CS, Zielinski R, Jayaraman A, Evers LJ, Foschini L, Mariakakis A, Pandey G, Shawen N, Synder P, Omberg L, BEAT-PD DREAM Challenge Consortium (2023). Developing better digital health measures of Parkinson's disease using free living data and a crowdsourced data analysis challenge. PLOS Digit Health.

[9] Lo C, Arora S, Baig F, Lawton MA, El Mouden C, Barber TR, Ruffmann C, Klein JC, Brown P, Ben-Shlomo Y, de Vos M, Hu MT (2019). Predicting motor, cognitive and functional impairment in Parkinson's. Ann Clin Transl Neurol.

[10] (2021). Recommendation on the ethics of artificial intelligence. United Nations Educational, Scientific and Cultural Organization.

[11] (2021). Ethics and governance of artificial intelligence for health. World Health Organization.

[12] Fröhlich H, Bontridder N, Petrovska-Delacréta D, Glaab E, Kluge F, Yacoubi ME, Marín Valero M, Corvol JC, Eskofier B, Van Gyseghem JM, Lehericy S, Winkler J, Klucken J (2022). Leveraging the potential of digital technology for better individualized treatment of Parkinson's disease. Front Neurol.

[13] (2020). Assessment List for Trustworthy Artificial Intelligence (ALTAI) for self-assessment. European Commission.

[14] Riggare S, Stamford J, Hägglund M (2021). A long way to go: patient perspectives on digital health for Parkinson's disease. J Parkinsons Dis.

[15] Badr J, Motulsky A, Denis JL (2024). Digital health technologies and inequalities: a scoping review of potential impacts and policy recommendations. Health Policy.

[16] Sendra A, Grosjean S, Bonneville L (2022). Co-constructing experiential knowledge in health: the contribution of people living with Parkinson to the co-design approach. Qual Health Commun.

[17] Espay AJ, Bonato P, Nahab FB, Maetzler W, Dean JM, Klucken J, Eskofier BM, Merola A, Horak F, Lang AE, Reilmann R, Giuffrida J, Nieuwboer A, Horne M, Little MA, Litvan I, Simuni T, Dorsey ER, Burack MA, Kubota K, Kamondi A, Godinho C, Daneault JF, Mitsi G, Krinke L, Hausdorff JM, Bloem BR, Papapetropoulos S (2016). Technology in Parkinson's disease: challenges and opportunities. Mov Disord.

[18] Grosjean S, Ciocca JL, Gauthier-Beaupré A, Poitras E, Grimes D, Mestre T (2022). Co-designing a digital companion with people living with Parkinson's to support self-care in a personalized way: the eCARE-PD Study. Digit Health.

[19] Laurisz N, Ćwiklicki M, Żabiński M, Canestrino R, Magliocca P (2023). Co-creation in health 4.0 as a new solution for a new era. Healthcare (Basel).

[20] Middel CN, Blake MR, Boelsen-Robinson T, Mackenbach JD, Stuber JM, Vargas C, Forrester-Bowling T (2024). Co-creation in public health research: an introduction to basic principles. Public Health Res Pract.

[21] Pérez Jolles M, Willging CE, Stadnick NA, Crable EL, Lengnick-Hall R, Hawkins J, Aarons GA (2022). Understanding implementation research collaborations from a co-creation lens: recommendations for a path forward. Front Health Serv.

[22] Couture V, Roy MC, Dez E, Laperle S, Bélisle-Pipon JC (2023). Ethical implications of artificial intelligence in population health and the public's role in its governance: perspectives from a citizen and expert panel. J Med Internet Res.

[23] Okun S, Hanger M, Browne-James L, Montgomery T, Rafaloff G, van Delden JJ (2023). Commitments for ethically responsible sourcing, use, and reuse of patient data in the digital age: cocreation process. J Med Internet Res.

[24] Ozkaynak M, Sircar CM, Frye O, Valdez RS (2021). A systematic review of design workshops for health information technologies. Informatics.

[25] Lundell S, Toots A, Sönnerfors P, Halvarsson A, Wadell K (2022). Participatory methods in a digital setting: experiences from the co-creation of an eHealth tool for people with chronic obstructive pulmonary disease. BMC Med Inform Decis Mak.

[26] Dugstad J, Eide T, Nilsen ER, Eide H (2019). Towards successful digital transformation through co-creation: a longitudinal study of a four-year implementation of digital monitoring technology in residential care for persons with dementia. BMC Health Serv Res.

[27] Monje MH, Grosjean S, Srp M, Antunes L, Bouça-Machado R, Cacho R, Domínguez S, Inocentes J, Lynch T, Tsakanika A, Fotiadis D, Rigas G, Růžička E, Ferreira J, Antonini A, Malpica N, Mestre T, Sánchez-Ferro Á (2023). Co-designing digital technologies for improving clinical care in people with Parkinson's disease: what did we learn?. Sensors (Basel).

[28] Revenäs Å, Hvitfeldt Forsberg H, Granström E, Wannheden C (2018). Co-designing an eHealth service for the co-care of Parkinson disease: explorative study of values and challenges. JMIR Res Protoc.

[29] Kessler D, Hauteclocque J, Grimes D, Mestre T, Côtéd D, Liddy C (2019). Development of the Integrated Parkinson's Care Network (IPCN): using co-design to plan collaborative care for people with Parkinson's disease. Qual Life Res.

[30] AI-PROGNOSIS.

[31] Tessarolo F, Petsani D, Conotter V, Nollo G, Conti G, Nikolaidou M, Onorati G, Bamidis PD, Konstantinidis EI (2022). Developing ambient assisted living technologies exploiting potential of user-centred co-creation and agile methodology: the CAPTAIN project experience. J Ambient Intell Humaniz Comput.

[32] Creswell JW, Creswell JD (2017). Research Design: Qualitative, Quantitative, and Mixed Methods Approaches.

[33] Braun V, Clarke V (2006). Using thematic analysis in psychology. Qual Res Psychol.

[34] McHugh ML (2012). Interrater reliability: the kappa statistic. Biochem Med (Zagreb).

[35] Janesick VJ (2007). Peer debriefing. The Blackwell Encyclopedia of Sociology.

[36] Asan O, Bayrak AE, Choudhury A (2020). Artificial intelligence and human trust in healthcare: focus on clinicians. J Med Internet Res.

[37] Habbal A, Ali MK, Abuzaraida MA (2024). Artificial intelligence trust, risk and security management (AI TRiSM): frameworks, applications, challenges and future research directions. Expert Syst Appl.

[38] Reinhardt K (2022). Trust and trustworthiness in AI ethics. AI Ethics.

[39] Balasubramaniam N, Kauppinen M, Rannisto A, Hiekkanen K, Kujala S (2023). Transparency and explainability of AI systems: from ethical guidelines to requirements. Inf Softw Technol.

[40] Ha T, Kim S (2023). Improving trust in AI with mitigating confirmation bias: effects of explanation type and debiasing strategy for decision-making with explainable AI. Int J Hum Comput Interact.

[41] Adadi A, Berrada M (2018). Peeking inside the black-box: a survey on explainable artificial intelligence (XAI). IEEE Access.

[42] Saeed W, Omlin C (2023). Explainable AI (XAI): a systematic meta-survey of current challenges and future opportunities. Knowl Based Syst.

[43] Godoy Junior CA, Miele F, Mäkitie L, Fiorenzato E, Koivu M, Bakker LJ, Groot CU, Redekop WK, van Deen WK (2024). Attitudes toward the adoption of remote patient monitoring and artificial intelligence in Parkinson's disease management: perspectives of patients and neurologists. Patient.

[44] Sun W, Nasraoui O, Shafto P (2020). Evolution and impact of bias in human and machine learning algorithm interaction. PLoS One.

[45] Abràmoff MD, Tarver ME, Loyo-Berrios N, Trujillo S, Char D, Obermeyer Z, Eydelman MB, Maisel WH, Foundational Principles of Ophthalmic Imaging and Algorithmic Interpretation Working Group of the Collaborative Community for Ophthalmic Imaging Foundation, Washington, D.C. (2023). Considerations for addressing bias in artificial intelligence for health equity. NPJ Digit Med.

[46] Dzialas V, Doering E, Eich H, Strafella A, Vaillancourt D, Simonyan K, van Eimeren T (2024). Houston, we have AI problem! Quality issues with neuroimaging-based artificial intelligence in Parkinson's disease: a systematic review. Mov Disord.

[47] Chen Z (2023). Ethics and discrimination in artificial intelligence-enabled recruitment practices. Humanit Soc Sci Commun.

[48] Laux J (2024). Institutionalised distrust and human oversight of artificial intelligence: towards a democratic design of AI governance under the European Union AI Act. AI Soc.

[49] Coiera E (2019). On algorithms, machines, and medicine. Lancet Oncol.

[50] Azencott CA (2018). Machine learning and genomics: precision medicine versus patient privacy. Philos Trans A Math Phys Eng Sci.

[51] Schaeffer E, Rogge A, Nieding K, Helmker V, Letsch C, Hauptmann B, Berg D (2020). Patients' views on the ethical challenges of early Parkinson disease detection. Neurology.

[52] De Freitas J, Agarwal S, Schmitt B, Haslam N (2023). Psychological factors underlying attitudes toward AI tools. Nat Hum Behav.

[53] Paccoud I, Valero MM, Marín LC, Bontridder N, Ibrahim A, Winkler J, Fomo M, Sapienza S, Khoury F, Corvol JC, Fröhlich H, Klucken J (2024). Patient perspectives on the use of digital medical devices and health data for AI-driven personalised medicine in Parkinson's disease. Front Neurol.

[54] Dias SB, Jelinek HF, Hadjileontiadis LJ (2024). Wearable neurofeedback acceptance model for students' stress and anxiety management in academic settings. PLoS One.

[55] Mittermaier M, Raza MM, Kvedar JC (2023). Bias in AI-based models for medical applications: challenges and mitigation strategies. NPJ Digit Med.

[56] Alves B, Mota PR, Sineiro D, Carmo R, Santos P, Macedo P, Carreira JC, Madeira RN, Dias SB, Pereira CM (2024). MoveONParkinson: developing a personalized motivational solution for Parkinson's disease management. Front Public Health.

[57] Macedo P, Madeira RN, Santos PA, Mota P, Alves B, Pereira CM (2024). A conversational agent for empowering people with Parkinson’s disease in exercising through motivation and support. Appl Sci.

