# Supplementary material for: Stakeholder Perspectives on Trustworthy AI for Parkinson Disease Management Using a Cocreation Approach: Qualitative Exploratory Study
Source: J Med Internet Res. 2025 Aug 6;27:e73710. doi: 10.2196/73710 (PMC12368464; doi:10.2196/73710)
Supplement: Multimedia Appendix 1 [file jmir_v27i1e73710_app1.docx]

# This is a Multimedia Appendix to a full manuscript published in the J Med Internet Res. For full copyright and citation information see <https://www.jmir.org/2025/1/e73710/>

## Script: Co-creation Workshop#1 with People with Parkinson's Disease (PwPD)

1. ***Trustworthy AI design (Be as concrete and specific as possible! Give examples!)***
2. What makes an AI system relating to health data…
   1. … trustworthy?
   2. … untrustworthy?
3. ***Human Autonomy in healthcare and selfcare (Be as concrete and specific as possible! Give examples!)***
4. Would you like to be informed about the involvement of AI in your healthcare decisions? If so, how?
5. Would knowing that a human healthcare professional is overseeing the AI's decisions affect your level of comfort? How?
6. In which situations would it be important for a human to have the final say, even if AI is used in the process?
7. ***Fairness and Equality (Be as concrete and specific as possible! Give examples!)***
8. How ould you define fairness in AI?
9. How can potential biases in healthcare delivery be addressed?
10. ***Explainability and Transparency (Be as concrete and specific as possible! Give examples!)***
11. Is it important to know how the AI makes predictions?
12. Would understanding how the AI works make you trust its predictions more?
13. What would transparency regarding how the AI makes decisions mean to you?
14. ***Other Considerations***
15. Are there any other considerations you believe should be prioritized when AI is used in healthcare?

## Script: Co-creation Workshop#2 with Healthcare professionals (HCPs)

1. ***Understanding Clinical Needs***
2. What are the most critical challenges you face in diagnosing and/or managing PD?
3. How can AI-enabled solutions that estimate PD risk and prognosis fit in modern PD care and clinical practice?
4. ***Utility of the prediction models***
5. PD risk model: How could an AI-driven PD risk prediction model assist you in early intervention and preventive strategies? What features or risk factors should the model prioritize to be most beneficial in a clinical setting?
6. AI's Role in PD Progression Monitoring: How would you like AI to assist in monitoring the progression of PD, and what specific indicators would be most valuable to track?
7. Medication Response Prediction: In what ways do you think an AI model predicting patient response to medication could improve treatment outcomes for PD?
8. ***Trust, Explainability and Transparency***
9. What factors would help you trust AI predictions regarding PD risk and prognosis?
10. How much information about the AI’s internal function would you need to feel comfortable using it in clinical practice? What level of detail would you need?
11. Imagine you use an AI-based software that provides estimations of PD risk for a person over time. How would you ideally like each risk estimation to be explained to you by the software?
12. ***Data Reliability and Quality***
13. What, if any, concerns do you have about the reliability/accuracy and quality of the data used to train AI models for PD?
14. How can these concerns be mitigated?
15. In what ways do you think that data used for the development of AI for PD risk estimation and prognosis can be biased?
16. What are some variables that we should give particular attention to, to identify potential biases, or conversely for which variables we should ensure that AI models perform equally (e.g., age, sex)?
17. ***Impact on Patient-Doctor Relationship and Clinical Practice***
18. How do you think the integration of AI in clinical decision-making will affect the patient-doctor relationship?
19. Do you worry that patients might prioritize AI-driven recommendations over your professional judgment? [If yes, what features should relevant AI tools have to avoid or mitigate that?]
20. Do you worry that patients may not take your opinion as seriously if they know you relied on AI assistance? [If yes, what features should relevant AI tools have to avoid or mitigate that?]
21. How can we ensure that AI enhances rather than undermines your role as a healthcare provider?
22. In a scenario where AI for PD diagnosis support and prognosis is used in clinical practice, are you concerned about overreliance (of healthcare professionals) on those ΑΙ tools? [If yes, what features should those tools have to avoid or mitigate that?]
23. ***Responsibility and Accountability***
24. How should responsibility be attributed when decisions supported by AI lead to poor or harmful patient outcomes?
25. What guidelines or protocols would you suggest to clearly define accountability for decisions made with AI support in healthcare scenarios?

1. ***Ethical Considerations***
2. Are there any other ethical concerns you foresee with the use of AI in predicting PD risk or progression or response to medication?
3. How should these concerns be addressed?
4. ***Anything else you would like to comment or discuss?***
